# Combined flexor sheath and gastrocnemius aponeurosis flap for the treatment of chronic calcified Achilles tendon rupture: a case report

**DOI:** 10.3389/fsurg.2025.1687970

**Published:** 2025-11-21

**Authors:** Shuangqiang Tu, Zhongshan Li, Mengying Xiao, Wensheng Zhu, Yi Shao

**Affiliations:** 1 College of Acupuncture and Orthopedics, Hubei University of Chinese Medicine, Wuhan, China; 2 Department of Osteology, Huanggang Hospital of Traditional Chinese Medicine Affiliated to Hubei University of Chinese Medicine, Huanggang, China; 3 School of Medicine, Hubei Minzu University, Enshi, China; 4 Department of Surgery, Huanggang Hospital of Traditional Chinese Medicine Affiliated to Hubei University of Chinese Medicine, Huanggang, China

**Keywords:** Achilles tendon, calcification, chronic rupture, suture anchor, case report

## Abstract

**Introduction:**

The Achilles tendon is the largest tendon in the human body and is prone to rupture when subjected to excessive dorsiflexion trauma of the ankle joint. The primary goal of treatment is to restore limb function; however, there remains considerable debate regarding the optimal management strategy.

**Patient concerns:**

We report a patient presenting with chronic Achilles tendon rupture accompanied by calcification of the tendon, which caused persistent pain and functional limitation of the affected limb.

**Diagnosis:**

Chronic Achilles tendon rupture with associated tendon calcification, with a tendon defect measuring approximately 6 cm on preoperative imaging, confirmed by clinical examination and imaging evaluation.

**Interventions:**

The patient underwent surgical removal of the calcified lesion, followed by reconstruction of the tendon defect using a gastrocnemius aponeurosis flap combined with the Achilles tendon sheath.

**Outcomes:**

Postoperative recovery was favorable, and at the 6-month follow-up, the patient achieved satisfactory ankle function with an AOFAS score of 89, a VISA-A score of 91, and an ATRS score of 90. At the 12-month follow-up, functional outcomes remained stable, with an AOFAS score of 95, a VISA-A score of 96, and an ATRS score of 94, confirming sustained recovery and tendon integrity.

**Conclusion:**

This case highlights a feasible surgical strategy for chronic Achilles tendon rupture with calcification, which may provide an alternative approach for tendon reconstruction in patients with sheath proliferation and calcified lesions.

## 1 Introduction

Achilles tendon rupture is a common tendon injury that is usually easy to diagnose; nevertheless, previous studies have documented a misdiagnosis rate as high as 25% (1). Early misdiagnosis or missed diagnosis may progress to chronic injury, which significantly increases the difficulty of treatment and worsens the prognosis, thereby causing considerable distress to patients' daily life and work (2). Chronic Achilles tendon rupture complicated with calcification is relatively rare in clinical practice (3). In this case, we performed calcified lesion debridement combined with suture anchor fixation, gastrocnemius aponeurosis flap, and Achilles tendon sheath reconstruction. This approach utilizes proliferative Achilles tendon sheath tissue as an autologous material, avoiding the need for distant tendon graft harvesting and thereby reducing donor-site morbidity. By combining the gastrocnemius aponeurosis flap with the thickened sheath, this technique provides sufficient mechanical strength for defect repair while minimizing additional trauma. The case is reported as follows.

## 2 Case presentation

The patient was an 18-year-old male who developed left ankle pain after falling to the ground following a jump while playing basketball on June 20, 2024. He initially sought care at a local hospital, where he was diagnosed with a “ankle sprain” and treated with plaster immobilization. After three weeks, the patient removed the plaster by himself and continued weight-bearing ambulation with a limp. Given the patient's young age, the presence of marked tendon calcification was considered unusual; possible causes such as previous minor injury, local chronic inflammation, or metabolic factors were taken into account.

On August 15, 2024, he presented to our hospital due to worsening pain in the left ankle. Physical examination revealed mild swelling of the left heel without obvious erythema, ulceration, or abnormal skin temperature. There was localized tenderness on the posterior aspect of the left ankle and slightly limited heel-raising movement.

**Figure 1:**
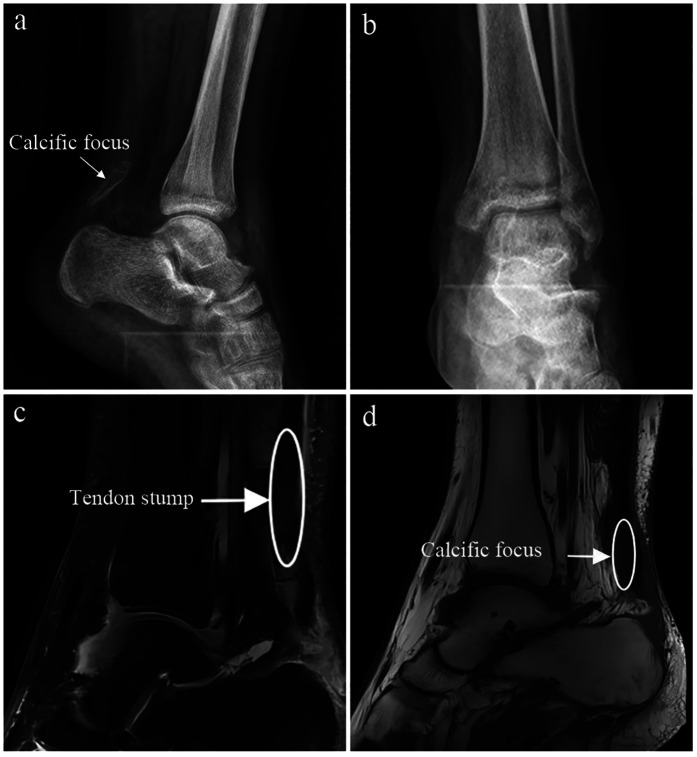
**(a–d)** Preoperative x-ray and magnetic resonance imaging (MRI) showing chronic rupture and defect of the left Achilles tendon with calcification.

Imaging studies, including x-ray and MRI, demonstrated thickening of the left Achilles tendon with intratendinous calcification and tendon rupture, with a tendon gap measuring approximately 6 cm as shown on preoperative MRI. Based on these findings, the diagnosis of chronic rupture of the left Achilles tendon with tendon defect and calcification was established (Figures 1a–d). Surgical treatment was recommended.

## 3 Treatment

**Figure 2:**
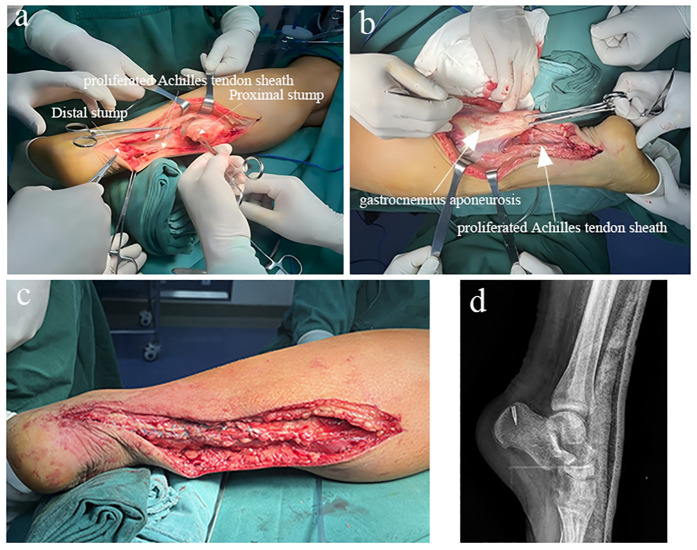
**(a,b)** Intraoperative exploration showing intact gastrocnemius aponeurosis and achilles tendon sheath with marked scar hyperplasia, and shortening of the Achilles tendon stumps. **(**c) The gastrocnemius aponeurosis flap and proliferated Achilles tendon sheath were folded and sutured into a cord-like structure resembling the native Achilles tendon. **(d**) Postoperative digital radiograph demonstrating appropriate placement of the suture anchor.

Three days after admission, the patient underwent “debridement of calcified lesion of the left Achilles tendon combined with suture anchor fixation, reconstruction with gastrocnemius aponeurosis flap and Achilles tendon sheath, and plaster immobilization.” Intraoperative exploration revealed that the gastrocnemius aponeurosis and Achilles tendon sheath were structurally intact but exhibited marked fibrotic thickening. The Achilles tendon was shortened, with an approximately 6-cm defect between the proximal and distal stumps (Figures 2a,b). The calcified lesion was located at the distal stump and demonstrated indistinct demarcation from the adjacent tendon tissue. After complete excision of the calcified portion, the tendon sheath was evaluated and found to be sufficiently thick and mechanically robust to serve as autologous reinforcement material. A gastrocnemius aponeurosis flap measuring approximately 8 cm in length and 3 cm in width, together with a thickened Achilles tendon sheath of about 6 cm in length, was harvested and prepared for reconstruction. The aponeurosis flap was then reversed and longitudinally interwoven with the thickened tendon sheath to reconstruct the defect. The construct was anchored to the calcaneal tuberosity using a suture anchor (Figures 2c,d). This approach was selected because it provided adequate coverage and tensile support without the need for distant tendon graft harvesting. Alternative options, including peroneus brevis transfer and allograft tendon reconstruction, were considered but deemed unnecessary given the adequate local tissue quality. The proximal and distal tendon stumps were re-approximated under appropriate tension and secured with a modified Kessler suture technique. Intraoperative passive ankle motion demonstrated smooth force transmission of the reconstructed tendon without excessive tension.

Postoperatively, the affected limb was immobilized in plantarflexion with a short-leg plaster cast for 4 weeks, followed by neutral (90° dorsiflexion) immobilization for 2 weeks. During the immobilization period, the patient was instructed to perform active toe flexion–extension exercises to promote venous return and prevent stiffness. After cast removal at 6 weeks, supervised physiotherapy was initiated, including ankle range-of-motion training and isometric strengthening exercises. Partial weight-bearing ambulation was permitted at 8 weeks postoperatively, progressing to full weight-bearing by 12 weeks as tolerated. Between 10 and 12 weeks, proprioceptive and balance training were introduced using a balance board and resistance bands. From 3 to 6 months, progressive strengthening of the gastrocnemius–soleus complex and endurance training were conducted. By 6 months postoperatively, the patient achieved full weight-bearing walking and could perform single-leg heel raises, indicating satisfactory functional recovery. The detailed rehabilitation schedule is summarized in Table 1.

**Table 1:** Postoperative rehabilitation timeline after Achilles tendon reconstruction.

Postoperative period	Management/Activity	Objectives
0–4 weeks	Immobilization in plantarflexion with short-leg plaster cast; active toe flexion–extension exercises	Protect repair site, promote circulation, prevent stiffness
4–6 weeks	Immobilization at neutral (90° dorsiflexion) position	Gradual tendon adaptation and tension balance
6–8 weeks	Initiate ankle range-of-motion and isometric strengthening under supervision	Restore controlled joint motion and early muscle activation
8–12 weeks	Progress from partial to full weight-bearing; introduce proprioceptive and balance training using resistance bands or balance board	Enhance stability, coordination, and muscle endurance
3–6 months	Progressive strengthening of the gastrocnemius–soleus complex; endurance and gait training; single-leg heel raises	Achieve full functional recovery and independent ambulation

**Figure 3:**
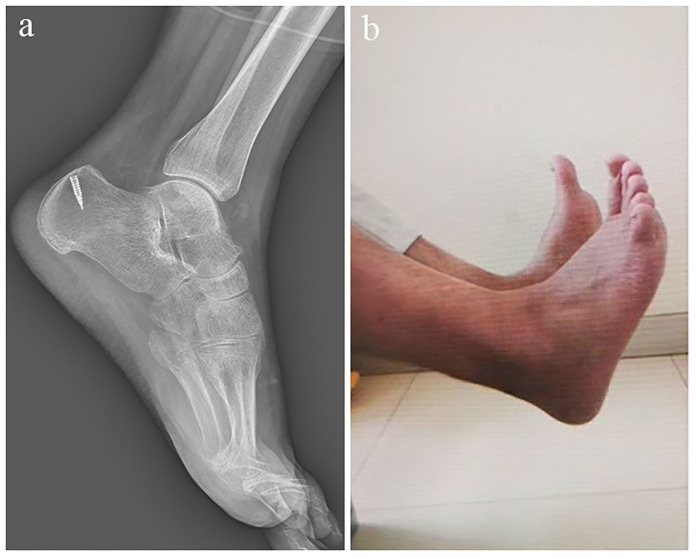
**(a,b)** At 6 months postoperatively, the patient demonstrated satisfactory recovery of ankle flexion and extension function.

At the 6-month follow-up, the patient showed restoration of ankle flexion and extension function (Figures 3a,b), with an AOFAS score of 89, a VISA-A score of 91, and an ATRS score of 90, indicating good short-term recovery. At the 12-month follow-up, the patient demonstrated stable and sustained improvement, achieving an AOFAS score of 95, a VISA-A score of 96, and an ATRS score of 94, with no pain, weakness, or limitation in daily activities. Follow-up ultrasonography confirmed continuous tendon integrity and satisfactory remodeling at the reconstruction site, suggesting durable functional outcomes.

## 4 Discussion

The Achilles tendon is the largest tendon in the human body, and rupture may occur when direct trauma or sudden excessive tensile force exceeds its structural limit. Due to the compensatory action of the posterior tibial muscle group, some patients with Achilles tendon rupture may still retain ankle flexion and extension, which often leads to underestimation by patients themselves and insufficient physical examination by clinicians. These are the main reasons for misdiagnosis and missed diagnosis.

For chronic Achilles tendon rupture, conservative treatment is generally unsatisfactory, and surgical intervention is usually required (4). Various surgical techniques have been described, with the choice of procedure largely determined by the size of the tendon defect (5). In recent years, minimally invasive techniques have been increasingly applied in Achilles tendon repair (6). However, concerns remain regarding the tensile strength of sutures in minimally invasive settings, and freshening of tendon ends in chronic ruptures is technically challenging under minimally invasive approaches (7). Allograft tendons and synthetic grafts have been reported as alternatives for reconstructing Achilles tendon defects, but their reliability requires further clinical validation (8). Autologous tendon or fascial grafting provides dependable biomechanical strength, yet is associated with greater donor-site morbidity. In the present case, the Achilles tendon defect measured approximately 6 cm. For defects of this magnitude, fascial or tendon transfer remains a commonly employed and effective reconstructive method (9).

Among the established surgical techniques, V–Y tendon advancement and flexor hallucis longus (FHL) tendon transfer are two widely recognized options. V–Y advancement, typically used for small to moderate defects, allows direct lengthening of the proximal tendon stump but may be limited by reduced tendon elasticity and proximal weakness (10). In contrast, FHL transfer provides a strong and vascularized autologous tendon for reconstruction, offering reliable biomechanical strength and promoting early rehabilitation (11–13). However, it involves sacrificing a functional toe flexor, which can result in loss of great toe plantarflexion strength. In addition to these methods, the use of gastrocnemius aponeurosis or paratenon tissue has been reported as an effective alternative for chronic Achilles tendon reconstruction. Recent studies have demonstrated that gastrocnemius aponeurosis flaps can provide sufficient mechanical strength and favorable biological integration for medium-to-large tendon defects while avoiding donor-site morbidity (14, 15). Similarly, the paratenon, when thickened or proliferative, offers an autologous vascularized tissue layer that enhances tendon gliding and healing (16). These reports collectively support the feasibility of utilizing local tissues in reconstruction, which aligns with the technique described in our case. Compared with these methods, our approach utilizes locally proliferated Achilles tendon sheath tissue in combination with a gastrocnemius aponeurosis flap, thereby preserving donor tendon function and reducing iatrogenic morbidity while maintaining adequate mechanical strength for repair.

Compared with conventional autologous or allograft tendon transfers, the current technique demonstrates several advantages. First, by utilizing locally proliferated Achilles tendon sheath tissue together with a gastrocnemius aponeurosis flap, this approach eliminates the need for distant tendon harvesting and consequently reduces donor-site morbidity. Second, the combination of fibrotic sheath tissue and aponeurosis provides adequate tensile strength and biological continuity, facilitating graft integration while minimizing additional trauma. Third, the reconstruction preserves the native anatomical alignment and maintains physiological tension of the Achilles tendon, which may contribute to faster functional recovery. Nevertheless, this technique has several limitations. This report describes a single case, which restricts the generalizability of the findings. Although the 12-month follow-up demonstrated favorable short-term outcomes, it remains insufficient to evaluate the long-term integrity and functional durability of the reconstructed tendon. Notably, postoperative ultrasonography or magnetic resonance imaging was not performed to confirm structural tendon integration. Future prospective studies with larger sample sizes, longer follow-up periods, and imaging-based assessments are warranted to further validate the efficacy and durability of this technique.

To minimize postoperative scar formation and adhesion, meticulous intraoperative tissue handling was emphasized to preserve the paratenon and reduce unnecessary soft-tissue trauma. Postoperatively, early functional rehabilitation—including controlled range-of-motion exercises after cast removal—was implemented to prevent adhesion formation and stiffness. In addition, scar management measures such as gentle massage, silicone gel application, and regular physiotherapy were employed to promote tissue remodeling and maintain tendon gliding. These steps were essential for optimizing functional recovery after such extensive dissection.

In summary, this case provides several important clinical insights. First, some patients with Achilles tendon rupture may present with atypical symptoms, and retained ankle motion can contribute to misdiagnosis. Therefore, careful clinical examination is crucial. The heel-rise test and Thompson (Simmonds–Thompson) calf-squeeze test provide high specificity, and when rupture is suspected, MRI or ultrasonography should be performed to ensure accurate evaluation and reduce missed diagnoses (17). Second, although the Achilles tendon sheath (paratenon) is typically too thin to serve as graft material, proliferative thickening of the sheath—as observed in this patient—may offer a valuable autologous source for reconstruction. This technique obviates the need for distant autograft harvesting, thereby reducing donor-site morbidity and iatrogenic trauma. Third, once a chronic Achilles tendon rupture is recognized, early surgical intervention is recommended to prevent progressive calcification and further enlargement of the tendon defect. Finally, the possible association between tendon sheath proliferation and calcification remains hypothetical and should be interpreted with caution; future studies with histopathological and molecular investigations are warranted to clarify this potential mechanism.

## 5 Conclusion

This report describes a patient with chronic Achilles tendon rupture complicated by intratendinous calcification. Intraoperatively, marked proliferation of the Achilles tendon sheath was observed. Although the potential relationship between tendon sheath proliferation and tendon calcification remains unclear, the proliferated sheath provided valuable autologous tissue for reconstruction. In combination with a gastrocnemius aponeurosis flap, it allowed for robust repair of the tendon defect. While this single case is not representative, it offers valuable insight into surgical options under such specific conditions and provides evidence-based guidance for treatment strategies in similar anatomical scenarios.

## Data Availability

The original contributions presented in the study are included in the article/Supplementary Material, further inquiries can be directed to the corresponding authors.
